# Methoxydiphenylamine-substituted fluorene derivatives as hole transporting materials: role of molecular interaction on device photovoltaic performance

**DOI:** 10.1038/s41598-017-00271-z

**Published:** 2017-03-10

**Authors:** Robertas Tiazkis, Sanghyun Paek, Maryte Daskeviciene, Tadas Malinauskas, Michael Saliba, Jonas Nekrasovas, Vygintas Jankauskas, Shahzada Ahmad, Vytautas Getautis, Mohammad Khaja Nazeeruddin

**Affiliations:** 1 Department of Organic Chemistry, Kaunas University of Technology, Radvilenu pl. 19, 50254 Kaunas, Lithuania; 2 Group for Molecular Engineering of Functional Materials, Institute of Chemical Sciences and Engineering, École Polytechnique Fédérale de Lausanne, Rue de l’Industry 17, CH-1951 Sion, Switzerland; 3 Department of Solid State Electronics, Vilnius University, Sauletekio 9, 10222 Vilnius, Lithuania; 4 Abengoa Research, C/Energía Solar n° 1, Campus Palmas Altas, 41014 Sevilla, Spain

## Abstract

The molecular structure of the hole transporting material (HTM) play an important role in hole extraction in a perovskite solar cells. It has a significant influence on the molecular planarity, energy level, and charge transport properties. Understanding the relationship between the chemical structure of the HTM's and perovskite solar cells (PSCs) performance is crucial for the continued development of the efficient organic charge transporting materials. Using molecular engineering approach we have constructed a series of the hole transporting materials with strategically placed aliphatic substituents to investigate the relationship between the chemical structure of the HTMs and the photovoltaic performance. PSCs employing the investigated HTMs demonstrate power conversion efficiency values in the range of 9% to 16.8% highlighting the importance of the optimal molecular structure. An inappropriately placed side group could compromise the device performance. Due to the ease of synthesis and moieties employed in its construction, it offers a wide range of possible structural modifications. This class of molecules has a great potential for structural optimization in order to realize simple and efficient small molecule based HTMs for perovskite solar cells application.

## Introduction

Hybrid organic-inorganic perovskite materials have shown great potential for use not only in photovoltaics but also in lasers, plasmonics, light-emitting diodes, tandems with silicon, photodetectors and sensors^1–4^. Recently, perovskite solar cells (PSCs) have attracted considerable attention due to extremely large and rapid performance progress. In 2009, Kojima *et al*. demonstrated that methylammonium lead iodide (MAPbI_3_) perovskite can work as a solar cell material with 3.8% power conversion efficiency (PCE)^5^. Since then published values leaped to 21.6%^6^ in only five years with a currently certified record PCE of 22.1%^7^. Such values are already exceeding commercialized polycrystalline silicon solar cells and rapidly approach crystalline silicon solar cells with record efficiency around 25.6%^8^.

In the PSC perovskite light absorber layer, either with or without mesoporous scaffold, is positioned between the electron and hole transport layers. By precisely manipulating charge carriers along the entire pathway from the absorber to both electrodes, an increase in the power conversion efficiency could be achieved. Although research is conducted on each layer, the biggest progress has been made in the area of perovskite film processing and relevant material design. Several reports show stability, performance and reproducibility improvements for the perovskite materials using mixtures of multiple cations, i.e. methylammonium, formamidinium and Cs, instead of single methylammonium^9, 10^. Tuning the halide composition, by using an increased amount of bromide instead of iodide, has also been reported to improve thermal and humidity stability^11^.

Although a number of new hole transporting materials (HTM) has been developed and investigated^12^, the field is still dominated by costly 2,2′,7,7′-tetrakis(*N*,*N*-di-*p*-methoxyphenylamine)-9-9′-spirobifluorene (Spiro-OMeTAD) and even more expensive poly[bis(4-phenyl)(2,4,6-trimethylphenyl)amine] (PTAA)^13–15^. The main factors contributing to the high cost of these materials are multistep synthesis procedures, expensive reagents and costly purification methods used^16^. For example, sublimation-grade Spiro-OMeTAD is needed in order to obtain high-performance devices. A significant effort has been made to develop less costly and easier to synthesize HTMs with performance comparable or better than that of Spiro-OMeTAD or PTAA^17–21^. One promising avenue for simplification of the HTM synthesis procedure is substitution of the difficult to obtain 9,9′-spirobifluorene core with a simpler alternative. Methoxydiphenylamine-substituted fluorene and triphenylamine derivatives have demonstrated their effectiveness as HTMs for number of applications and they form a basis of two most effective HTMs used for PSC construction, Spiro-OMeTAD and PTAA^6, 15, 18, 22^. Therefore, it’s appropriate to test the combination of the two as a new class of HTMs for application in PSCs. Additionally, it was demonstrated that structure of the HTMs has a significant influence on the molecular planarity, energy level and charge transport properties^23^. Understanding the relationship between the chemical structure of the HTMs and the photovoltaic performance is crucial in continued development of the more efficient organic charge transporting materials and is imperatively needed^24^. Numerous investigations are being carried out aimed at enhancement of hole drift mobility; however, until now only a few tangible recommendations have been made with regard to molecular structure modifications^25–27^.

**Figure 1:**
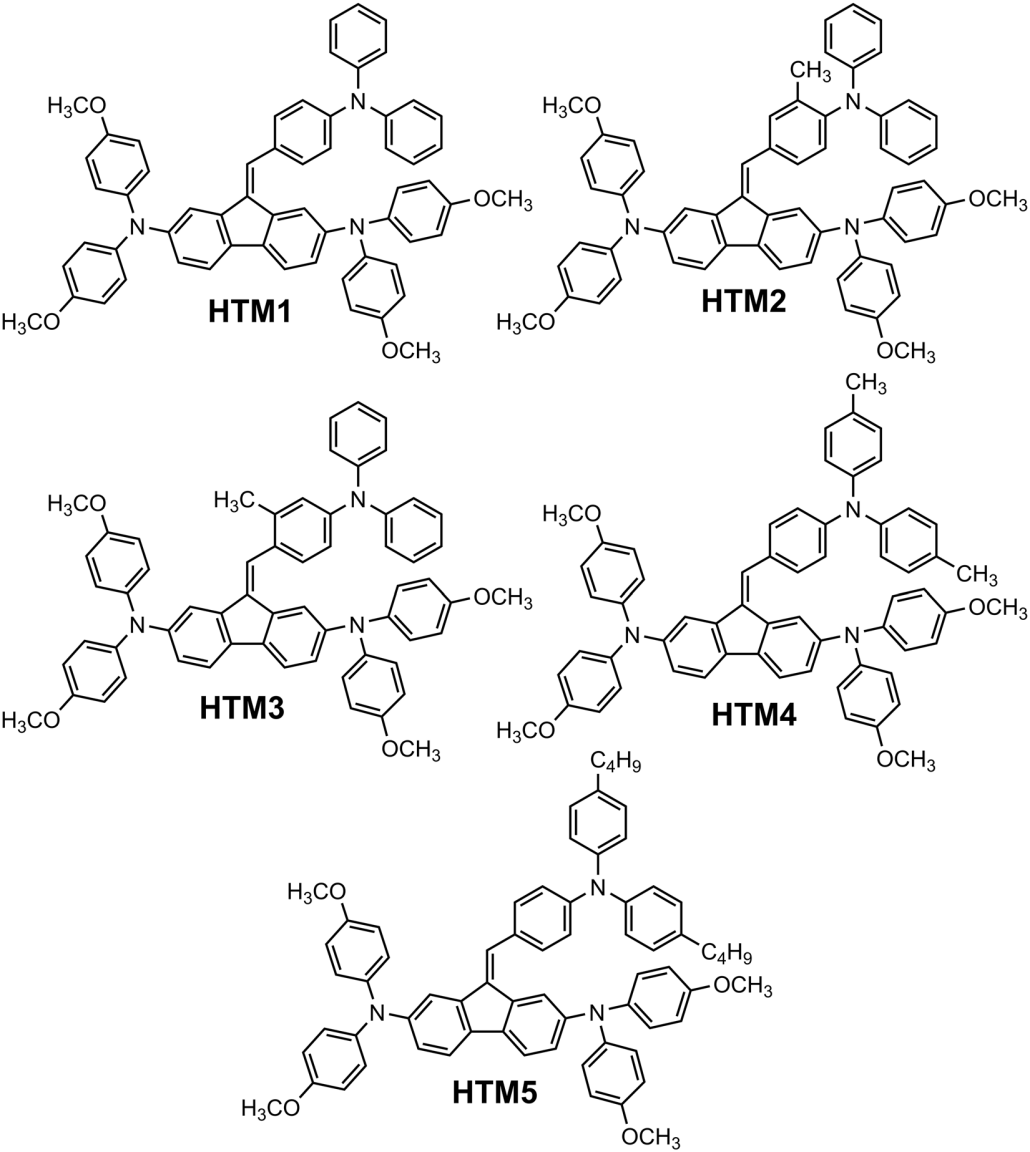
Structures of the investigated hole transporting materials **HTM1–5**.

In this report a new type of HTMs (Fig. 1), based on methoxydiphenylamine-substituted fluorene and triphenylamine fragments is reported. Using molecular engineering approach, *i.e.* influencing conformation and packing of the molecules in the film *via* changing angle of rotation between fluorene and triphenylamine moieties with the help of additional methyl groups or by placing different aliphatic substituents at the *para* positions of the triphenylamine fragments, we have constructed a series of HTMs in order to investigate the relationship between the chemical structure of the HTMs and the photovoltaic performance. PCEs in the range of 9% to 16.8% was measured utilizing these HTMs, highlighting the importance of the optimal chemical structure, as an inappropriately placed methyl group could compromise device performance.

## Results

**Figure 2:**
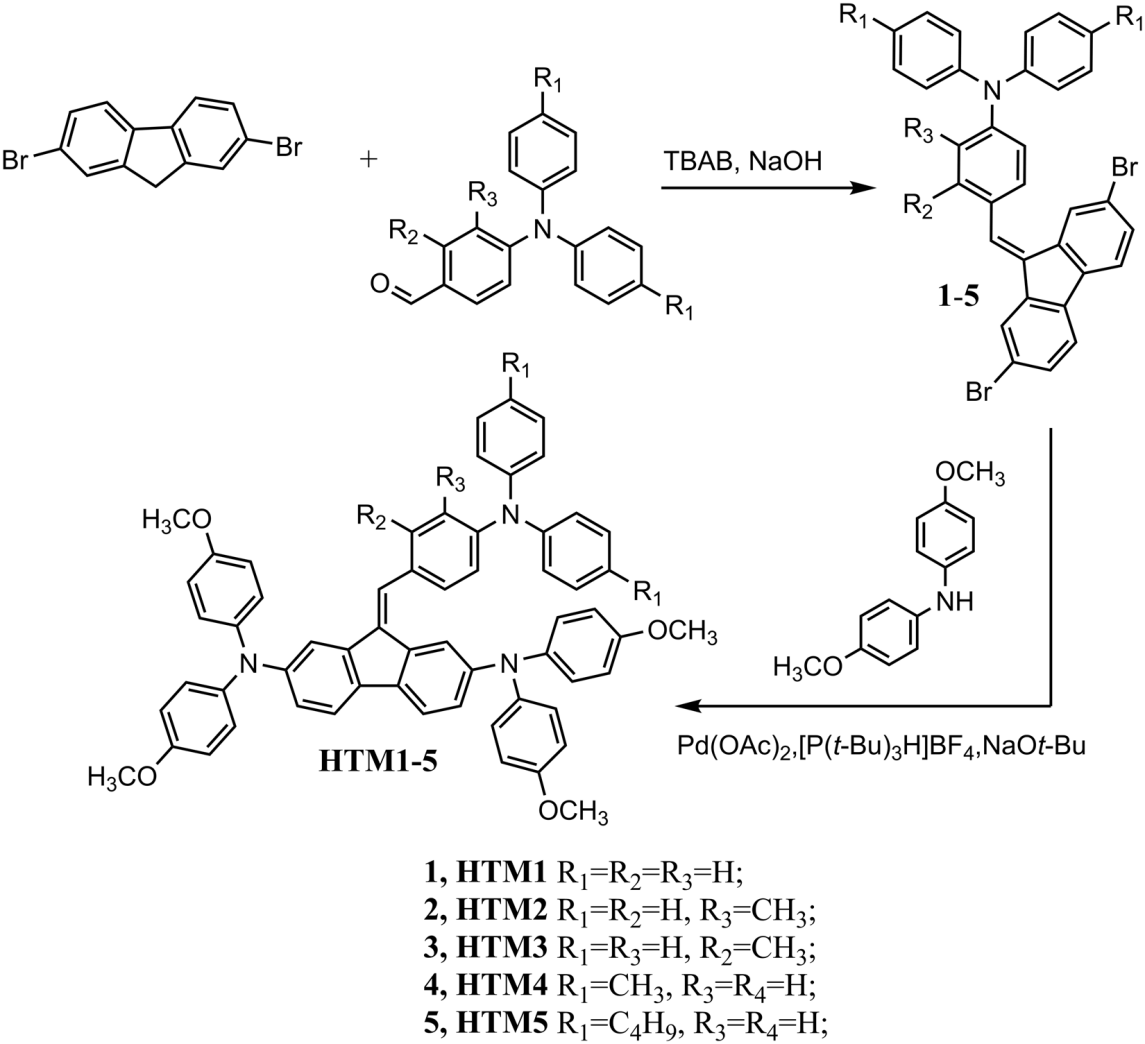
Synthetic route to hole transporting materials **HTM1–5**.

Synthesis of new type of hole transporting molecules involves condensation of 2,7-dibromofluorene with corresponding formyltriphenylamine, followed by a palladium-catalysed C–N cross coupling reaction with 4,4′-dimethoxydiphenylamine (Fig. 2). More detailed information on the synthesis can be found in the Supporting Information.

**Figure 3:**
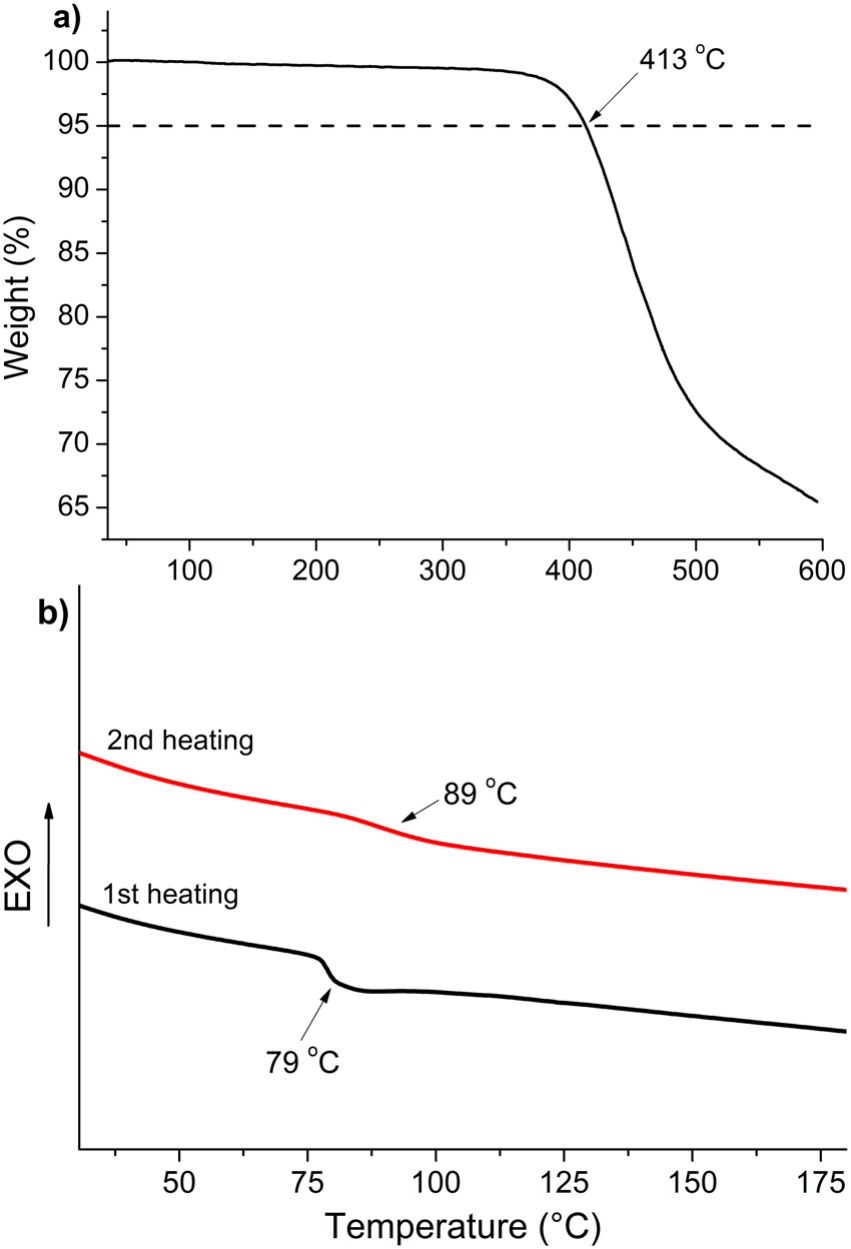
Thermogravimetric (**a**) and differential scanning calorimetry (**b**) heating curves of **HTM4** (heating rate 10 K min^−1^, N_2_ atmosphere).

Thermogravimetric analysis (TGA) reveals very good thermal stability of the investigated HTMs, the fluorene derivatives start to decompose at temperatures above 410 °C (Fig. 3a, Table 1 and Supplementary Figs S1–S4).

**Table 1:** Thermal and optical properties of the Spiro-OMeTAD, **HTM1–5**.

Compound	*T*_*m*_ (°C)	*T*_*g*_^a^ (°C)	*T*_*dec*_ (°C)	Abs_max_^b^ (nm)	*ε* (M^−1^ cm^−1^)
HTM1	—	108	416	382	5.5 × 10^4^
HTM2	—	104	419	383	5.8 × 10^4^
HTM3	—	104	418	383	5.5 × 10^4^
HTM4	—	89	413	383	5.2 × 10^4^
HTM5	—	85	413	385	5.4 × 10^4^
Spiro	245	126	449	387	6.9 × 10^4^

^a^Determined by DSC: scan rate, 10 K min^−1^; N_2_ atmosphere; second run. ^b^Measured in 10^−4^ M THF solution.

During differential scanning calorimetry (DSC) scans it was determined that investigated materials are fully amorphous. Only the glass to liquid transition is detected and it is in the range between 85 and 109 °C (Fig. 3b, Table 1 and Supplementary Figs S5–S8). This is desired in order to form homogeneous HTM films in SC devices. *T*_g_ of **HTM1–3** are similar to that of Spiro-OMeTAD (126 °C), while presence of aliphatic substituents in *para* positions of the tripenylamine fragment in **HTM4** and **HTM5** reduce the glass transition temperature by ~20 °C.

**Figure 4:**
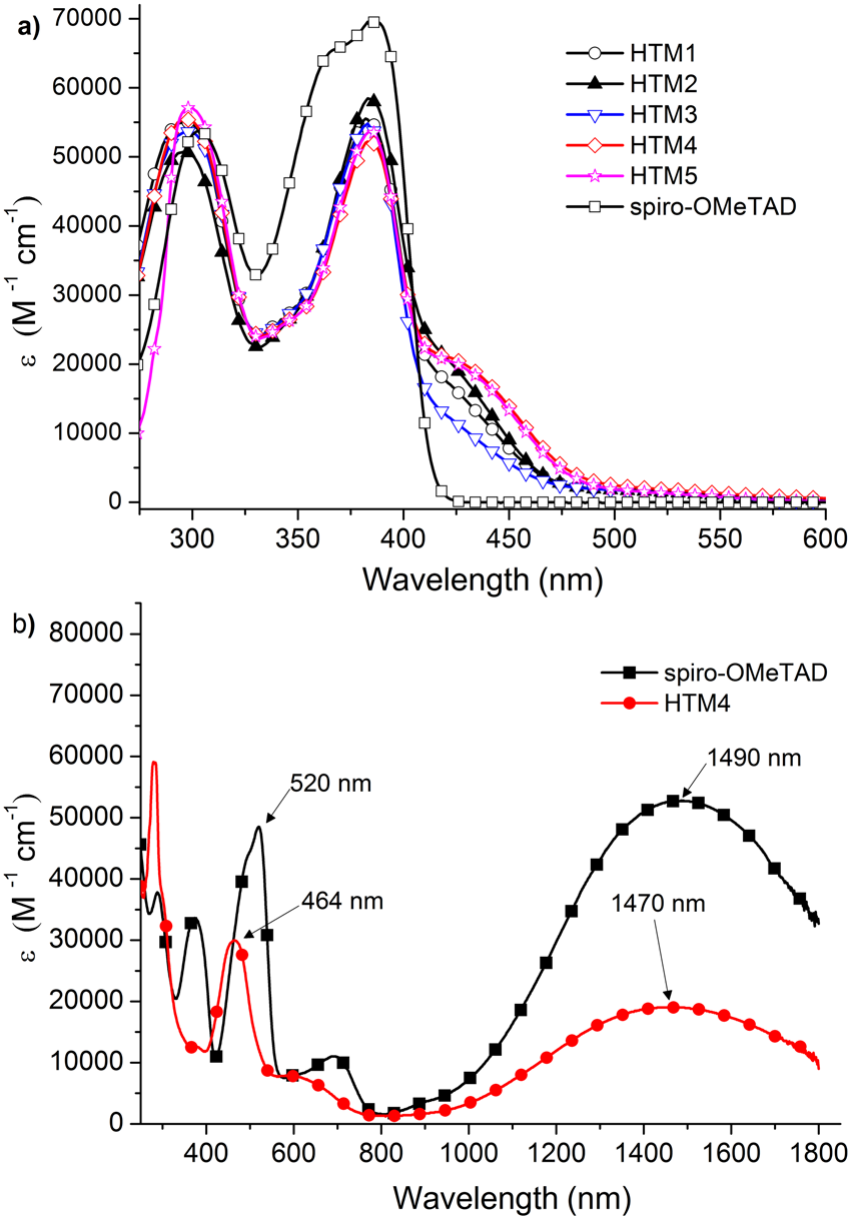
UV–vis absorption spectra of **HTM1–5**, Spiro-OMeTAD (**a**) and oxidized Spiro(TFSI)_2_, HTM4TFSI (**b**) in THF (c = 10^−4^ M).

The UV-vis absorption bands of the investigated **HTM1–5** are shown in Fig. 4a. All studied derivatives are based on the same principal structure, *i.e.* fluorene core with attached 4,4′-dimethoxydiphenylamine fragments connected with a triphenylamine unit; therefore, their UV-vis spectra bear significant similarities.

Presence of a fluorene fragment with attached 4,4′-dimethoxydiphenylamine moieties, the same as in Spiro-OMeTAD, results in a comparable UV-Vis absorption spectra. The π–π* absorption bands for **HTM1–5** are observed at around 383 nm, indicating a similar sized π–conjugated system as that of Spiro-OMeTAD. Presence of the triphenylamine fragment results in the increase in size of the π–conjugated system and an additional absorption shoulder at 430 nm is observed^28^. Position of the methyl group in the triphenylamine segment has a noticeable influence on the intensity of the shoulder. Methyl group in the *meta* position of the triphenylamine fragment in **HTM3** causes it to be more twisted out of the plain. As a consequence shoulder at 430 nm is less intense, compared with other investigated HTMs, indicating that methyl group at this position restricts π–electron conjugation in the molecule and limits the ability of the π–electrons of triphenylamine moiety to join the common system. On the other hand, alkyl groups at *para* positions of the triphenylamine moiety (**HTM4**, **HTM5**) have a positive effect on the intensity of the absorption band at 430 nm. UV-Vis-NIR absorbance spectra, upon chemical oxidation of **HTM4** and Spiro-OMeTAD by the silver bis(trifluoromethanesulfonyl)imide (AgTFSI)^29^, are shown in the Fig. 4b.

Similarly as Spiro(TFSI)_2_, chemically oxidized HTM4TFSI demonstrated absorption bands at around 460 nm, 650 nm and 1470 nm indicating formation of the oxidized species. The lower intensity of the HTM4TFSI absorption maxima could be attributed to the fact that the transition is accompanied by a smaller change in the electronic charge distribution upon excitation as **HTM1–5** have one less methoxydiphenylamine-substituted fluorene fragment available for oxidation.

Cyclic voltammetry (CV) was used to determine the ground-state oxidation potentials of the HTMs (Table 2). Quasi-reversible oxidation signals are observed for all investigated materials (Supplementary Figs S12–16) and differences in the CV results are quite small, although, there are some slight variations in the energy levels. A slight increase of *E*_HOMO_, compared with **HTM1**, is observed for **HTM2**, **HTM4**, **HTM5** due to presence of additional alkyl groups and stronger interactions between methoxydiphenylamine-substituted fluorene and triphenylamine fragments. **HTM3** on the other hand, doesn’t follow a similar pattern as methyl group in the *meta* position of the triphenylamine moiety restricts π–electron conjugation in the molecule, these results correlate well with the UV-vis absorption data.

**Table 2:** Electrochemical characteristics, *I*_p_, hole mobility for Spiro-OMeTAD, **HTM1–5**.

Compound	*E*_HOMO_^a^ (eV)	*I*_p_^b^ (eV)	*μ*_0_^c^ (cm^2^ V^−1^ s^−1^)	*μ* ^d^ (cm^2^ V^−1^ s^−1^)
HTM1	−5.13	5.05	1.4 × 10^−5^	5.9 × 10^−4^
HTM2	−5.05	5.00	1.3 × 10^−5^	3.8 × 10^−4^
HTM3	−5.14	5.00	1 × 10^−9^	3 × 10^−7^
HTM4	−5.05	4.92	2.2 × 10^−5^	3.8 × 10^−4^
HTM5	−5.08	5.03	1.1 × 10^−5^	3.8 × 10^−4^
Spiro	−5.12	5.00	4.1 × 10^−5^	5 × 10^−4^
HTM1 + PC	—	—	3.9 × 10^−7^	2.3 × 10^−5^
HTM2 + PC	—	—	4.0 × 10^−7^	1.9 × 10^−5^
HTM3 + PC	—	—	1.5 × 10^−8^	1.3 × 10^−7^
HTM4 + PC	—	—	1.1 × 10^−6^	3.7 × 10^−5^
HTM5 + PC	—	—	5.8 × 10^−7^	2.8 × 10^−5^
Spiro + PC	—	—	2.9 × 10^−6^	6.8 × 10^−5^

^a^CV measurements were carried out at a glassy carbon electrode in dichloromethane solutions containing 0.1 M tetrabutylammonium hexafluorophosphate as electrolyte and Pt wire as the reference electrode. Each measurement was calibrated with ferrocene (Fc). Conversion factors: ferrocene in DCM vs SCE 0.46^34^, SCE vs SHE: 0.244^35^, SHE vs. vacuum: 4.43^36^. ^b^Ionization potential was measured by the photoemission in air method from films. ^c^Mobility value at zero field strength. ^d^Mobility value at 6.4 × 10^5^ V cm^−1^ field strength.

The solid-state ionization potential (*I*_p_), was measured by photoelectron spectroscopy in air (PESA) method in the films of the undoped HTMs (Supplementary Figs S9 and S10 and Table 2). The structure/energy level dependency is less clearly defined in tightly packed films compared with the solvated molecules. Measured *I*_p_ values are slightly lower than the HOMO levels found in the CV experiments and the difference may arise from different measurement methods and conditions (solution in CV and solid film in the photoemission method) used. Overall from the ionization potential data presented in the Table 2 it can be stated that *I*_p_ values of the HTMs containing alkyl substituents in the triphenylamine fragment are lower as compared to the parent derivative **HTM1.** The most noticeable decrease in *I*_p_ is observed for **HTM4** with two methyl groups in *para* positions of the triphenylamine moiety. PESA and CV measurements reveal that solid-state *I*_p_ and oxidation potentials in solution of the investigated compounds are similar to those of the Spiro-OMeTAD, as all of them share the same methoxydiphenylamine-substituted fluorene main fragment.

Charge-transporting properties of the investigated HTMS were determined by the xerographic time-of-flight (XTOF) technique. Supplementary Fig. S17a demonstrates representative dU/dt hole-transients for the thin film of **HTM3**. It exhibits a dispersive hole-transport; which, along with the strong electric-field mobility dependence, suggests the trap-dominant charge transport in this material. Nevertheless, well-defined transit times (*t*_t_) established from the intersection points of two asymptotes of double-logarithmic plots provided hole-drift mobility at respective applied fields. Similar performance was also established for **HTM2**, **HTM4**, and **HTM5**. Hole transport of **HTM1** is Gaussian and transit time is defined in linear plot of transients (Supplementary Fig. S17b). Examples of mobility field dependencies are given in the Supplementary Fig. S11. In all cases investigated mobility *μ* may be well approximated by the formula:

$$\mu ={\mu }_{0}\exp (\alpha \sqrt{E})$$

here *μ*_0_ is the zero field mobility, *α* is Pool-Frenkel parameter and *E* is electric field strength. Such mobility dependence is explainable by terms of the Borsenberger and Weiss^30^, and Borsenberger *et al.* ^31^ disorder formalism. The mobility defining parameters *μ*_0_ and *α* values as well as the mobility value at the 6.4 × 10^5^ V cm^−1^ field are given in Table 2.

As seen from the results, synthesized HTMs demonstrated competitive charge mobility. XTOF measurements of the films prepared from **HTM1**, **HTM2**, **HTM4**, **HTM5** indicate that the hole-drift mobility is ~10^−5^ cm^2^ V^−1^s^−1^ at weak electric fields and ~3 × 10^−4^ cm^2^ V^−1^s^−1^ at a field strength of 6.4 × 10^5^ Vcm^−1^. The hole mobility of these HTMs is comparable to that of Spiro-OMeTAD, while **HTM3** demonstrates several orders of magnitude lower results. Most likely methyl group in the *meta* position of the triphenylamine fragment causes it to be more twisted out of the plain which prevents tight packing of the molecules and makes it more difficult for the charge to hop between sites.

**Figure 5:**
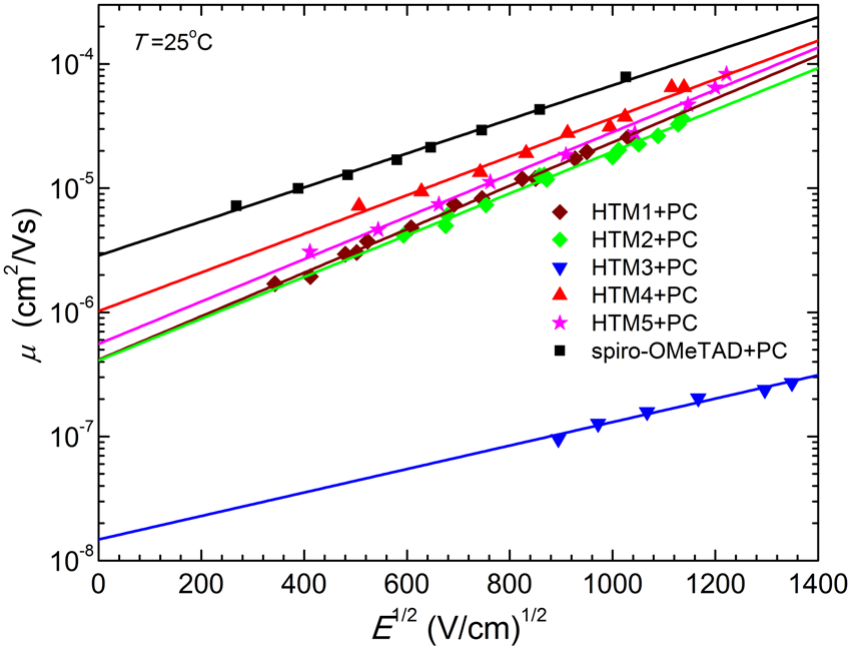
Electric-field dependencies of the hole drift mobilities in charge-transport layers of **HTM1–5** dispersed in polycarbonate polymeric matrix.

It is well known that quality of the prepared films can have significant impact on the results of mobility measurements^32^. Every molecule has different film forming properties, therefore in order to mitigate film quality influence on mobility results we have also performed XTOF measurements with HTMs dispersed in polycarbonate (PC) polymeric matrix which minimizes HTM molecule dependent film quality variations (Fig. 5 and Table 2). Naturally, absolute mobility values are lower due to the presence of large portion of nonconductive polymer; however structure/properties relation is much better expressed in this case. Methyl group in the *meta* position of the triphenylamine fragment in **HTM3** causes significant negative changes in the molecule’s conformation which translates into lowest charge mobility. While mobility in **HTM4**, **HTM5**, with alkyl groups at *para* positions of the triphenylamine moiety, is up to two orders of magnitude higher due to better optimized structure. XTOF measurement results in the HTM:polycarbonate mixtures also correlate well with the UV-Vis spectroscopy data.

The new semiconductors **HTM1–5** were tested as HTMs in perovskite solar cells using a device stack of fluorine doped tin oxide(FTO)/compact TiO_2_/mesoporous TiO_2_/perovskite/HTM/Au.

**Figure 6:**
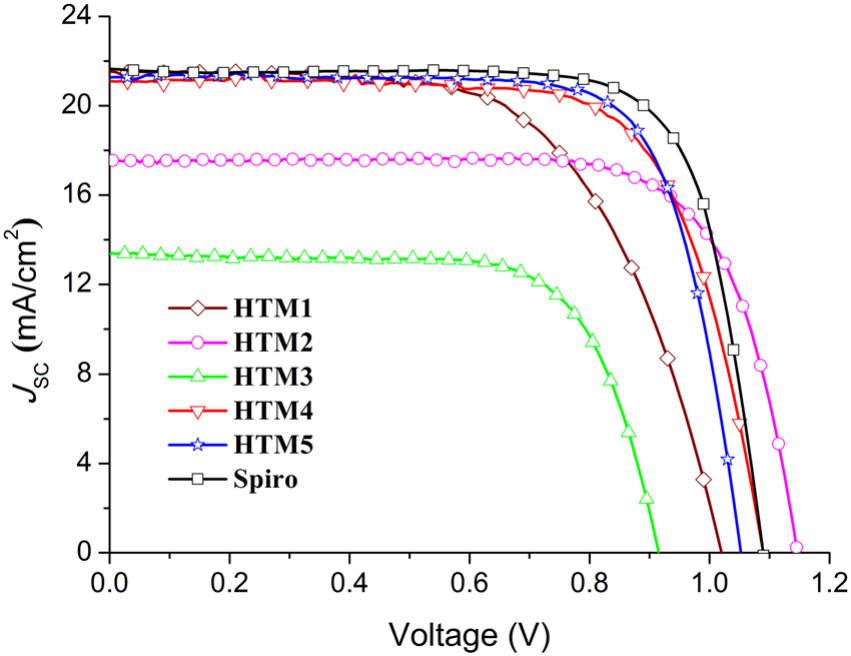
Current (*J*)-voltage (*V*) curves of the solar cells with **HTM1–5**, and **Spiro-OMeTAD** (control) recorded under AM 1.5 conditions (100 mW/cm^2^).

Position of the methyl groups in the molecule had a noticeable impact on the performance of the semiconductors in the PSC. Arrangement of the molecules into conformations less favourable for the charge transport in **HTM3** had a visible negative impact on charge mobility. Expectedly, decreased mobility had a negative effect on the performance of the HTM in the PSC as the PCE was only 9.15% (Fig. 6 and Supplementary Table S1). Change of the methyl group position from *meta* to *ortho* (**HTM2**) or its removal (**HTM1**) increases the mobility and also performance of the HTM in the PSC, PCE jumps to ~14–15%. The best results were obtained with structures containing aliphatic substituents in the *para* position of the triphenylamine moiety (**HTM4**, **HTM5**) which incidentally also demonstrated highest mobility in HTM:polycarbonate mixtures. PCE of 16.8% under AM 1.5 G illumination was recorded in the PSC with HTM4 (Table S1). The open-circuit voltage (*V*_oc_) was determined to be 1052 mV, current density (*J*_sc_) 21.3 mA cm^−2^ and fill factor 0.75 (Fig. 6). Similar PCE (16.5%) was also showed by **HTM5**. The best device prepared following the same device fabrication procedure, but using Spiro-OMeTAD as hole transporter, exhibited PCE of 17.88%.

## Conclusion

In conclusion, a new group of small-molecule hole transporting materials, based on methoxydiphenylamine-substituted fluorene and triphenylamine fragments, is demonstrated. These HTMs are synthesized in two steps from commercially available materials. Relationship between the chemical structure of the HTMs and the photovoltaic performance has been investigated using molecules with strategically placed aliphatic substituents. It was found that the structure of the HTMs play an important role in their hole extraction in PSCs and can have a significant influence on the molecular planarity, charge transport properties and device characteristics. We have observed that aliphatic substituents in *meta* position of the triphenylamine fragment cause it to be more twisted out of the plain and severely reduce charge transport properties of the HTM and overall device characteristics of the perovskite solar cells. In general, altering the structure of the phenyl ring connecting the fluorene and triphenylamine moieties produces undesirable conformational changes in the molecule and reduces its overall performance of the HTM in the PSC. On the other hand, positive effect on the performance of the hole transporting materials is observed after substitution at *para* position of the triphenylamine moiety. The overall efficiency of the investigated HTM in perovskite-based solar cells was in a range of 9% to 16.8% demonstrating the importance of the optimal molecular structure. Due to the ease of synthesis and moieties employed in its construction, it offers a wide range of possible structural modifications. The reported class of molecules has a great potential for further structure optimization in order to realize simple and efficient small molecular HTMs applied in perovskite solar cells.

## Methods

### Photovoltaic device preparation

Chemically eched FTO glass (Nippon Sheet Glass) was sequentially cleaned by sonication in a 2% Helmanex solution, acetone and ethanol for 30 min each, followed by a 15 min UV-ozone treatment. To form a 30 nm thick TiO_2_ blocking layer, diluted titanium diisopropoxide bis(acetylacetonate) (TAA) solution (Sigma-Aldrich) in isopropanol was sprayed at 450 °C. For the 200 nm mesoporous TiO_2_ layer, mesoporous-TiO_2_ layers were made by spin-coating a commercially available TiO_2_ paste (Dyesol 30NRD). Substrates were baked at 500 °C for 30 min. Then, Li-doping of mesoporous TiO_2_ is treated by spin coating a 0.1 M solution of Li-TFSI in acetonitrile at 3000 rpm for 10 s followed by another sintering at 500 °C for 20 min before the deposition of the perovskite layer. Mixed-perovskite precursor was prepared by mixing 1.15 m PbI_2_, 1.10 m FAI, 0.2 m PbBr_2_, 0.2 m MABr in a mixed solvent of DMF:DMSO = 4:1 (volume ratio). Perovskite solutions are successively spin-coated in the glovebox as follows: first, 2000 rpm for 10 s with a ramp-up of 200 rpm s^−1^; second, 6000 rpm for 30 s with a ramp-up of 2000 rpm s^−1^ ^33^. Chlorobenzene (CB, 100 μl) was dropped on the spinning substrate during the second spin-coating step 20 s before the end of the procedure. Films were annealed at 100 °C for 90 min. The hole-transporting materials were applied from a 60 mM solution in chlorobenzene. All HTMs were doped with bis(trifluoromethylsulfonyl)imide lithium salt (Li-TFSI, Sigma-Aldrich), tris(2-(1H-pyrazol-1-yl)-4-tert-butylpyridine)- cobalt(III) tris(bis(trifluoromethylsulfonyl)imide) (FK209, Dynamo) and 4-tert-Butylpyridine (TBP, Sigma-Aldrich). The molar ratio of additives for the HTMs where: 0.5, 0.03 and 3.3 for Li-TFSI, FK209 and TBP respectively. The HTM solutions were spin-coated onto the perovskite layers at 4000 rpm for 30 s. The gold electrodes were deposited by thermal evaporation of 80 nm gold in high vacuum conditions.

### Photovoltaic device testing

The solar cells were measured using a 450 W xenon light source (Oriel). The spectral mismatch between AM1.5 G and the simulated illumination was reduced by the use of a Schott K113 Tempax filter (Präzisions Glas & Optik GmbH). The light intensity was calibrated with a Si photodiode equipped with an IR-cutoff filter (KG3, Schott), and it was recorded during each measurement. Current-voltage characteristics of the cells were obtained by applying an external voltage bias while measuring the current response with a digital source meter (Keithley 2400). The voltage scan rate were 5 and 10 mV s^−1^ and no device preconditioning, such as light soaking or forward voltage bias applied for long time, was applied before starting the measurement. The starting voltage was determined as the potential at which the cells furnish 1 mA in forward bias, no equilibration time was used. The cells were masked with a black metal mask (0.16 cm^2^) to fix the active area and reduce the influence of the scattered light.

## Electronic supplementary material

Supplementary information

## References

[1] Mitzi, D. B. Synthesis, Structure, and Properties of Organic-Inorganic Perovskites and Related Materials In *Progress in Inorganic Chemistry* (ed. K. D. Karlin) Volume 48, 1–121 (John Wiley & Sons, 1999).

[2] Saliba M (2016). Structured organic–inorganic perovskite toward a distributed feedback laser. Adv. Mater..

[3] Saliba M (2015). Plasmonic-induced photon recycling in metal halide perovskite solar cells. Adv. Funct. Mater..

[4] Albrecht S (2016). Monolithic perovskite/silicon-heterojunction tandem solar cells processed at low temperature. Energy Environ. Sci..

[5] Kojima A, Teshima K, Shirai Y, Miyasaka T (2009). Organometal halide perovskites as visible-light sensitizers for photovoltaic cells. J. Am. Chem. Soc..

[6] Saliba M (2016). Incorporation of rubidium cations into perovskite solar cells improves photovoltaic performance. Science.

[7] NREL research cell record efficiency chart http://www.nrel.gov/pv/assets/images/efficiency_chart.jpg (accessed 2016. 11. 24).

[8] Masuko K (2014). Achievement of more than 25% conversion efficiency with crystalline silicon heterojunction solar cell. Ieee. J. Photovolt..

[9] Yi C (2016). Entropic stabilization of mixed A-cation ABX3 metal halide perovskites for high performance perovskite solar cells. Energy Environ. Sci..

[10] Li Z, Yang M, Park J-S, Wei S-H, Berry JJ, Zhu K (2016). Stabilizing perovskite structures by tuning tolerance factor: formation of formamidinium and cesium lead iodide solid-state alloys. Chem. Mater..

[11] Noh JH, Im SH, Heo JH, Mandal TN, Seok SI (2013). Chemical management for colorful, efficient, and stable inorganic–organic hybrid nanostructured solar cells. Nano Lett..

[12] Calio L, Kazim S, Grätzel M, Ahmad S (2016). Hole-transport materials for perovskite solar cells. Angew. Chem. Int. Ed..

[13] Yang WS (2015). Science.

[14] Yu Z, Sun L (2015). Recent progress on hole-transporting materials for emerging organometal halide perovskite solar cells. Adv. Energy Mater..

[15] Kim H, Lim K-G, Lee T-W (2016). Planar heterojunction organometal halide perovskite solar cells: roles of interfacial layers. Energy Environ. Sci..

[16] Saragi TPI, Spehr T, Siebert A, Fuhrmann-Lieker T, Salbeck J (2007). Spiro compounds for organic optoelectronics. Chem. Rev..

[17] Saliba M (2016). A molecularly engineered hole-transporting material for efficient perovskite solar cells. Nat. Energy.

[18] Bi D (2016). Facile synthesized organic hole transporting material for perovskite solar cell with efficiency of 19.8%. Nano Energy.

[19] Molina-Ontoria A (2016). Angew. Chem. Int. Ed..

[20] Zhang, J. *et al.* Constructive Effects of Alkyl Chains: A Strategy to Design Simple and Non-Spiro Hole Transporting Materials for High-Efficiency Mixed-Ion Perovskite Solar Cells. *Adv. Energy Mater*. 1502536 (2016).

[21] Li H (2014). Angew. Chem. Int. Ed..

[22] Shirota Y, Kageyama H (2007). Charge carrier transporting molecular materials and their applications in devices. Chem. Rev..

[23] Zhang, J. B. *et al.* Constructive effects of alkyl chains: a strategy to design simple and non-spiro hole transporting materials for high-efficiency mixed-ion perovskite solar cells. *Adv. Energy Mater*. 1502536 (2016).

[24] Salado, M. *et al.* Interface play between perovskite and hole selective layer on the performance and stability of perovskite solar cells. *ACS Appl. Mater. Interfaces*, 10.1021/acsami.6b12236 (2016). 10.1021/acsami.6b12236 27935300

[25] Jeon NJ (2014). o-Methoxy substituents in spiro-ometad for efficient inorganic–organic hybrid perovskite solar cells. J. Am. Chem. Soc..

[26] Rakstys K (2015). Triazatruxene-based hole transporting materials for highly efficient perovskite solar cells. J. Am. Chem. Soc..

[27] Tomkute-Luksiene D (2016). Molecular engineering of the hole-transporting material spiro-OMeTAD via manipulation of alkyl groups. RSC Adv..

[28] Lukes V (2007). Structure, electronic and optical characterization of oligothiophenes terminated with (9H-fluoren-9-ylidene)methyl chromophores. Synth. Met..

[29] Nguyen WH, Bailie CD, Unger EL, McGehee MD (2014). Enhancing the hole-conductivity of spiro-OMeTAD without oxygen or lithium salts by using spiro(TFSI)_2_ in perovskite and dye-sensitized solar cells. J. Am. Chem. Soc..

[30] Borsenberger, P. M. & Weiss D. S. *Organic Photoreceptors for Imaging Systems* (Marcel Dekker, 1993).

[31] Borsenberger PM, Pautmeier L, Bässler H (1991). Charge transport in disordered molecular solids. J. Chem. Phys..

[32] Bässler H, Köhler A (2012). Charge transport in organic semiconductors. Top. Curr. Chem..

[33] Bi D (2016). Efficient luminescent solar cells based on tailored mixed-cation perovskites. Sci. Adv..

[34] Connelly N, Geiger W (1996). Chemical redox agents for organometallic chemistry. Chem. Rev..

[35] Pavlishchuk V, Addison A (2000). Conversion constants for redox potentials measured versus different reference electrodes in acetonitrile solutions at 25 °C. Inorg. Chim. Acta.

[36] Reiss H, Heller A (1985). The absolute potential of the standard hydrogen electrode: a new estimate. J. Phys. Chem..

