# Supplementary material for: Methoxydiphenylamine-substituted fluorene derivatives as hole transporting materials: role of molecular interaction on device photovoltaic performance
Source: Sci Rep. 2017 Mar 10;7:150. doi: 10.1038/s41598-017-00271-z (PMC5428027; doi:10.1038/s41598-017-00271-z)
Supplement: Supplementary file 1 — Supplementary information [file 41598_2017_271_MOESM1_ESM.pdf]

Supplementary information for:

Methoxydiphenylamine-substituted fluorene derivatives as hole transporting materials: role of molecular interaction on device photovoltaic performance

Robertas Tiazkis[1], Sanghyun Paek[2], Maryte Daskeviciene[1], Tadas Malinauskas[1], Michael Saliba[2], Jonas Nekrasovas[3], Vygintas Jankauskas[3], Shahzada Ahmad[4], Vytautas Getautis[1,], Mohammad Khaja Nazeeruddin[2,]**

[1] Department of Organic Chemistry, Kaunas University of Technology, Radvilenu pl. 19, Kaunas, 50254, Lithuania

[2] Group for Molecular Engineering of Functional Materials, Institute of Chemical Sciences and Engineering, École Polytechnique Fédérale de Lausanne, Sion CH-1951, Switzerland

[3] Department of Solid State Electronics, Vilnius university, Sauletekio 9, Vilnius 10222, Lihuania

[4] Abengoa Research, C/Energía Solar n° 1, Campus Palmas Altas, 41014 Sevilla, Spain

General methods and materials

All reagents were purchased from commercial companies and used as received. The ^{1}H and ^{13}C NMR spectra were taken on Bruker Avance III 400 (400 MHz) spectrometer at RT. All the data are given as chemical shifts in δ (ppm). The course of the reactions products were monitored by TLC on ALUGRAM SIL G/UV254 plates and developed with UV light. Silica gel (grade 9385, 230–400 mesh, 60 Å, Aldrich) was used for column chromatography. Elemental analysis was performed with an Exeter Analytical CE-440 elemental analyser, Model 440 C/H/N/. Differential scanning calorimetry was performed on a Q10 calorimeter (TA Instruments) at a scan rate of 10 K min^{-1} in the nitrogen atmosphere. The glass transition temperatures for the investigated compounds were determined during the second heating scan. Thermogravimetric analysis was performed on a Q50 thermogravimetric analyser (TA Instruments) at a scan rate of 10 K min^{-1} in the nitrogen atmosphere. UV/Vis spectra were recorded on a PerkinElmer Lambda 35 spectrometer. UV-VIS-NIR spectra were recorded on a Shimadzu UV-3600 spectrophotometer. Microcells with an internal width of 1 mm were used. Melting points of the crystalline materials were determined using Electrothermal Mel-Temp DigiMelt MPA 161

melting point apparatus. IR-spectroscopy was performed on a Perkin Elmer Spectrum BX II FT-IR System, using KBr pellets.

Cyclic voltammetry measurements

Electrochemical studies were carried out by a three-electrode assembly cell and potentiostat-galvanostat from Bio-Logic SAS. Measurements were carried out with a glassy carbon electrode in dichloromethane solutions containing 0.1 M tetrabutylammonium hexafluorophosphate as electrolyte and Pt wire as the reference electrode, and a Pt wire counter electrode at a scan rate 50 mV s^{-1}. Each measurement was calibrated with ferrocene (Fc).

Ionization Potential Measurements

The solid state ionization potential (I_p) of the layers of the synthesized compounds was measured by the electron photoemission in air method.[1,2] The samples for the ionization potential measurement were prepared by dissolving materials in THF and were coated on Al plates pre-coated with ~0.5 μm thick methylmethacrylate and methacrylic acid copolymer adhesive layer. The thickness of the transporting material layer was 0.5-1 μm. Usually photoemission experiments are carried out in vacuum and high vacuum is one of the main requirements for these measurements. If vacuum is not high enough the sample surface oxidation and gas adsorption are influencing the measurement results. In our case, however, the organic materials investigated are stable enough to oxygen and the measurements may be carried out in the air. The samples were illuminated with monochromatic light from the quartz monochromator with deuterium lamp. The power of the incident light beam was (2-5)·10^{-8} W. The negative voltage of –300 V was supplied to the sample substrate. The counter-electrode with the 4.5×15 mm^2 slit for illumination was placed at 8 mm distance from the sample surface. The counter-electrode was connected to the input of the BK2-16 type electrometer, working in the open input regime, for the photocurrent measurement. The 10^{-15} – 10^{-12} A strong photocurrent was flowing in the circuit under illumination. The photocurrent I is strongly dependent on the incident light photon energy hν. The $I^{0.5} = f(h\nu)$ dependence was plotted. Usually the dependence of the photocurrent on incident light quanta energy is well described by linear relationship between $I^{0.5}$ and $h\nu$ near the threshold. The linear part of this dependence was extrapolated to the hν axis and I_p value was determined as the photon energy at the interception point.

Hole Drift Mobility Measurements

The samples for the hole mobility measurements were prepared by spin-coating the solutions of the compositions of synthesized compounds on polyester films with conductive Al layer. Polymer:HTM mixtures were prepared by dissolving corresponding HTM and polycarbonate (PC-Z) (Mitsubishi Gas Chemical Co., polycarbonate Iupilon Z-200) in 1:1 mass proportion in THF. The layer thickness was in the range of 2-6 μm. The hole drift mobility was measured by xerographic time of flight technique (XTOF).[3,4] Electric field was created by positive corona charging. The charge carriers were generated at the layer surface by illumination with pulses of nitrogen laser (pulse duration was 2 ns, wavelength 337 nm). The layer surface potential decrease as a result of pulse illumination was up to 1-5 % of initial potential before illumination. The capacitance probe that was connected to the wide frequency band electrometer measured the speed of the surface potential decrease dU/dt. The transit time t_t was determined by the kink on the curve of the dU/dt transient in double logarithmic scale. The drift mobility was calculated by the formula $\mu = d^2/U_0 t_t$, where d is the layer thickness, U_0 – the surface potential at the moment of illumination.

The detailed synthetic procedures:

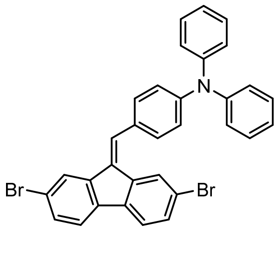

4-[(2,7-dibromo-9*H*-fluoren-9-ylidene)methyl]-*N*,*N*-diphenylaniline (1)

13 ml of 40% NaOH solution and tetrabuthylammonium bromide (0.58 g, 1.8 mmol) were added to a solution of 2,7-dibromofluorene (1.3 g 4 mmol) and 4-(diphenylamino)benzaldehyde (1.09 g, 4 mmol) in 13 ml of toluene. The reaction was heated at 100 °C for 10 minutes. After cooling, the resulting mixture was quenched with distilled water and extracted with ethyl acetate. The organic layer was dried over anhydrous Na_2SO_4, filtered, and the solvent was evaporated. The crude product was purified by column chromatography using 1:249 v/v acetone/*n*-hexane as an eluent to collect **1** as an orange solid. Yield 1.97 g (85 %) m.p.: 174–175.5 °C. ^{1}H NMR (400 MHz, $CDCl_3$): δ 7.89 (d, J = 1.5 Hz, 1H), 7.86

(d, $J = 1.5$ Hz, 1H), 7,62 (s, 1H), 7.54-7.07 (m, 18H). ^{13}C NMR (101 MHz, $CDCl_3$): δ 148.62, 147.18, 141.34, 138.81, 138.16, 136.67, 133.26, 131.07, 130.73, 130.54, 130.21, 129.46, 128.77, 127.27, 125.08, 123.68, 123.44, 122.28, 121.10, 120.97, 120.89, 120.66. Elemental analysis (calcd., found for $C_{32}H_{21}Br_2N$): C (66.34, 66.47), H (3.65, 3.84), N (2.42, 2.29).

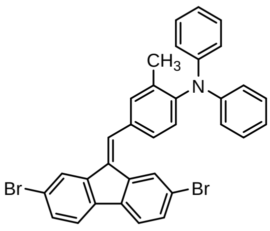

4-[(2,7-dibromo-9*H*-fluoren-9-ylidene)methyl]-2-methyl-*N*,*N*-diphenylaniline (2)

21 ml of 40% NaOH solution and tetrabuthylammonium bromide (0.41 g, 1.3 mmol) were added to a solution of 2,7-dibromofluorene (2.27 g 7 mmol) and 4-(diphenylamino)-3-methylbenzaldehyde (2.01 g, 7 mmol) in 21 ml of toluene. The reaction was heated at 100 °C for 10 minutes. After cooling, the resulting mixture was quenched with distilled water and extracted with ethyl acetate. The organic layer was dried over anhydrous Na_2SO_4, filtered, and the solvent was evaporated. The crude product was purified by column chromatography using 1:249 v/v acetone/*n*-hexane as an eluent to collect **2** as a yellow solid. Yield 3.41 g (82%) m.p.: 154–156 °C. ^{1}H NMR (400 MHz, $CDCl_3$): δ 7.91 (d, $J = 1.8$ Hz, 1H), 7.84 (d, $J = 1.8$ Hz, 1H), 7.74 (d, $J = 1.8$ Hz, 3H), 7.61-6.93 (m, 16H), 2.12, 1.57 (two s of rotamers, 3H). ^{13}C NMR (101 MHz, $CDCl_3$): δ 190.96, 148.27, 146.61, 144.73, 142.26, 141.41, 138.73, 138.20, 137.48, 136.58, 136.54, 135.27, 132.83, 131.93, 130.98, 130.65, 130.62, 130.53, 129.67, 129.30, 127.85, 127.61, 127.57, 127.16, 126.55, 123.39, 123.33, 122.99, 122.73, 121.86, 121.09, 120.96, 120.89, 120.65, 119.80, 18.70. Elemental analysis (calcd., found for $C_{33}H_{23}Br_2N$): C (66.80, 66.69), H (3.91, 4.04), N (2.36, 2.49).

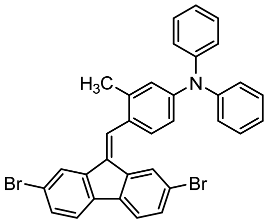

4-[(2,7-dibromo-9*H*-fluoren-9-ylidene)methyl]-3-methyl-*N*,*N*-diphenylaniline (3)

18 ml of 40% NaOH solution and tetrabuthylammonium bromide (0.35 g, 1.1 mmol) were added to a solution of 2,7-dibromofluorene (1.94 g 6 mmol) and 4-(diphenylamino)-2-methylbenzaldehyde (1.72 g, 6 mmol) in 18 ml of toluene. The reaction was heated at 100 °C for 10 minutes. After cooling, the resulting mixture was quenched with distilled water and extracted with ethyl acetate. The organic layer was dried over anhydrous Na_2SO_4, filtered, and the solvent was evaporated. The crude product was purified by column chromatography using 1:249 v/v acetone/*n*-hexane as an eluent to collect **3** as a yellow solid. Yield 2.35 g (66%) m.p.: 157–158.5 °C. ^{1}H NMR (400 MHz, $CDCl_3$): δ 7.92, 7.85, (two s of rotamers, 1H), 7.90 (s, 1H), 7.65, 7.61 (two s of rotamers, 1H), 7.54-6.90 (m, 18H), 2.33, 2.23 (two s of rotamers, 3H). ^{13}C NMR (101 MHz, $CDCl_3$): δ 148.76, 148.65, 147.50, 147.24, 147.10, 141.40, 140.90, 139.44, 138.82, 138.74, 138.50, 138.18, 136.96, 136.65, 134.34, 133.09, 131.12, 131.07, 130.94, 130.71, 130.60, 130.54, 130.34, 129.47, 129.41, 129.33, 128.86, 128.51, 127.56, 127.26, 125.90, 125.11, 124.79, 124.74, 124.71, 123.67, 123.62, 123.44, 123.24, 122.47, 122.07, 121.17, 121.13, 121.01, 120.98, 120.96, 120.92, 120.82, 120.69, 26.96, 21.51, 20.32. Elemental analysis (calcd., found for $C_{33}H_{23}Br_2N$): C (66.80, 66.65), H (3.91, 3.79), N (2.36, 2.31).

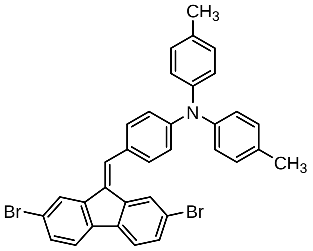

4-[(2,7-dibromo-9*H*-fluoren-9-ylidene)methyl]-*N*,*N*-bis(4-methylphenyl)aniline (4)

30 ml of 40% NaOH solution and tetrabuthylammonium bromide (0.58 g, 1.8 mmol) were added to a solution of 2,7-dibromofluorene (3.24 g 10 mmol) and 4-[bis(4-methylphenyl)amino]benzaldehyde (3.01 g, 10 mmol) in 30 ml of toluene. The reaction was heated at 100 °C for 10 minutes. After cooling,

the resulting mixture was quenched with distilled water and extracted with ethyl acetate. The organic layer was dried over anhydrous Na_2SO_4, filtered, and the solvent was evaporated. The crude product was purified by column chromatography using 1:249 v/v acetone/*n*-hexane as an eluent to collect **4** as a red solid. Yield 4.37 g (72%), m.p.: 162–163.5 °C. ^{1}H NMR (400 MHz, $CDCl_3$): δ 7.95 (d, *J* = 1.6 Hz, 1H), 7.82 (d, *J* = 1.6 Hz, 1H), 7.56 (s, 1H), 7.49-7.04 (m, 16H), 2.33 (s, 6H). ^{13}C NMR (101 MHz, $CDCl_3$): δ 149.05, 144.63, 141.49, 138.73, 138.20, 136.54, 133.55, 132.64, 130.95, 130.63, 130.56, 130.53, 130.15, 127.65, 127.17, 125.42, 123.36, 121.08, 120.96, 120.87, 120.84, 120.66, 26.97, 20.96. Elemental analysis (calcd., found for $C_{34}H_{25}Br_2N$): C (67.23, 67.10), H (4.15, 4.28), N (2.31, 2.46).

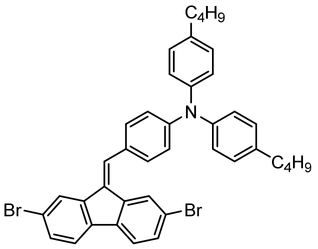

4-[(2,7-dibromo-9*H*-fluoren-9-ylidene)methyl]-*N*,*N*-bis(4-butylphenyl)aniline (5)

36 ml of 40% NaOH solution and tetrabuthylammonium bromide (0.58 g, 1.8 mmol) were added to a solution of 2,7-dibromofluorene (3.24 g 10 mmol) and 4-[bis(4-butylphenyl)amino]benzaldehyde (3.85 g, 10 mmol) in 36 ml of toluene. The reaction was heated at 100 °C for 10 minutes. After cooling, the resulting mixture was quenched with distilled water and extracted with ethyl acetate. The organic layer was dried over anhydrous Na_2SO_4, filtered, and the solvent was evaporated. The crude product was purified by column chromatography using 1:249 v/v acetone/*n*-hexane as an eluent to collect **5** as an orange solid. Yield 5.18 g (75%) m.p.: 75–76.5 °C. ^{1}H NMR (400 MHz, $CDCl_3$): δ 7.96 (d, *J* = 1.8 Hz, 1H), 7.86 (d, *J* = 1.8 Hz, 1H), 7.61 (s, 1H), 7.54-7.07 (m, 16H), 2.59 (t, *J* = 7.8 Hz, 4H), 1.60 (quin, *J* = 7.8 Hz, 4H), 1.38 (sex, *J* = 7.8 Hz, 4H), 0.95 (t, *J* = 7.8 Hz, 6H). ^{13}C NMR (101 MHz, $CDCl_3$): δ 149.09, 144.70, 141.49, 138.72, 138.57, 138.22, 136.55, 132.67, 130.93, 130.55, 129.37, 127.64, 127.20, 125.27, 123.36, 121.05, 120.93, 120.85, 120.64, 35.12, 33.67, 22.45, 14.00. Elemental analysis (calcd., found for $C_{40}H_{37}Br_2N$): C (69.47, 69.28), H (5.39, 5.43), N (2.03, 2.20).

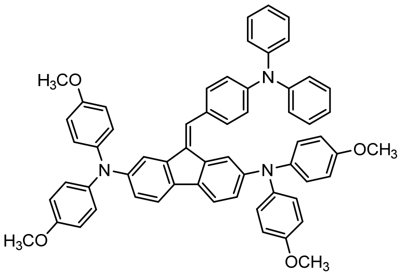

4-[{2,7-bis(4,4'-dimethoxydiphenylamino)-9*H*-fluoren-9-ylidene}methyl)-*N*,*N*-diphenylaniline (HTM1)

The mixture of **1** (1.16 g, 2 mmol), bis(4-methoxyphenyl)amine (1.38 g, 6 mmol), palladium acetate (0.009 g, 0.04 mmol), tri-tert-buthylphosphonium tetrafluoroborate (0.016 g, 0.054 mmol), sodium tert-butoxide (0.58 g, 6 mmol) in 13 ml of anhydrous toluene was refluxed for 52 hours under argon atmosphere. Afterwards, water was added and the extraction was done with ethyl acetate. The organic layer was dried over anhydrous Na_2SO_4, filtered, and the solvent was evaporated. The crude product was purified by column chromatography using 1:24 v/v tetrahydrofurane/*n*-hexane as an eluent to collect compound **HTM1** as an orange solid. The product was precipitated from 20% solution of the solid residue in acetone which was poured while intensively stirring into a twentyfold excess of ethanol. Yield: 1.40 g (80%). ^{1}H NMR (400 MHz, $CDCl_3$): δ 7.40-6.71 (m, 37H), 3.79 (s, 6H), 3.61 (s, 6H). ^{13}C NMR (101 MHz, $CDCl_3$): δ 147.65, 147.49, 141.10, 130.44, 129.36, 124.41, 123.90, 123.01, 119.57, 114.73, 55.63, 55.52. Elemental analysis (calcd., found for $C_{60}H_{49}N_3O_4$): C (82.26, 82.17), H (5.64, 5.51), N (4.80, 4.67).

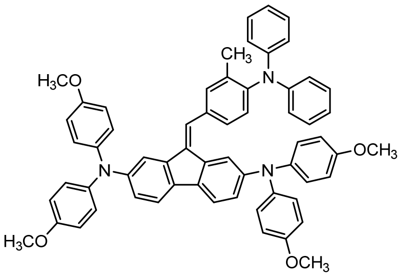

4-[{2,7-bis(4,4'-dimethoxydiphenylamino)-9*H*-fluoren-9-ylidene}methyl]-*N*,*N*-diphenyl-2-methylaniline (HTM2)

The mixture of **2** (2.37 g, 4 mmol), bis(4-methoxyphenyl)amine (2.75 g, 12 mmol), palladium acetate (0.018 g, 0.08 mmol), tri-tert-buthylphosphonium tetrafluoroborate (0.032 g, 0.11 mmol), sodium tert-butoxide (1.15 g, 12 mmol) in 26 ml of anhydrous toluene was refluxed for 52 hours under argon atmosphere. Afterwards, water was added and the extraction was done with ethyl acetate. The organic layer was dried over anhydrous Na_2SO_4, filtered, and the solvent was evaporated. The crude product was purified by column chromatography using 3:47 v/v tetrahydrofurane/*n*-hexane as an eluent to collect compound **HTM2** as an orange solid. The product was precipitated from 20% solution of the solid residue in acetone which was poured while intensively stirring into a twentyfold excess of ethanol. Yield: 2.56 g (72%). ^{1}H NMR (400 MHz, $CDCl_3$): δ 7.64 (s, 1H), 7.42-6.65 (m, 35H), 3.79 (s, 6H), 3.61 (s, 6H), 1.96 (s, 3H). ^{13}C NMR (101 MHz, $CDCl_3$): δ 147.17, 147.08, 145.05, 141.14, 137.56, 136.42, 131.78, 130.38, 129.55, 129.11, 128.85, 127.42, 126.16, 125.78, 121.90, 121.78, 120.92, 119.70, 114.61, 114.05, 55.52, 55.40, 18.63. Elemental analysis (calcd., found for $C_{61}H_{51}N_3O_4$): C (82.31, 82.39), H (5.78, 5.59), N (4.72, 4.55).

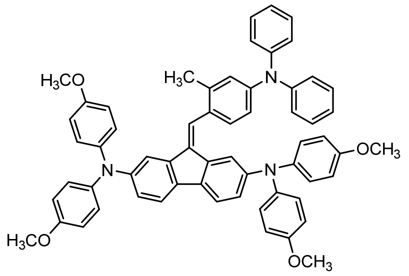

4-[{2,7-bis(4,4'-dimetoxydiphenylamino)-9*H*-fluoren-9-ylidene}methyl]-*N*,*N*-diphenyl-3-methylaniline (HTM3)

The mixture of **3** (1.19 g, 2 mmol), bis(4-methoxyphenyl)amine (1.38 g, 6 mmol), palladium acetate (0.009 g, 0.04 mmol), tri-tert-buthylphosphonium tetrafluoroborate (0.016 g, 0.054 mmol), sodium tert-butoxide (0.58 g, 6 mmol) in 13 ml of anhydrous toluene was refluxed for 52 hours under argon atmosphere. Afterwards, water was added and the extraction was done with ethyl acetate. The organic layer was dried over anhydrous Na_2SO_4, filtered, and the solvent was evaporated. The crude product was purified by column chromatography using 3:47 v/v tetrahydrofurane/*n*-hexane as an eluent to collect compound **HTM3** as an orange solid. The product was precipitated from 20% solution of the solid residue in acetone which was poured while intensively stirring into a twentyfold excess of ethanol. Yield: 0.68 g (57%). ^{1}H NMR (400 MHz, $CDCl_3$): δ 7.57 (d, J = 2.1 Hz, 1H), 7.41-6.54 (m, 35H), 3.79 (s, 6H), 3.62 (s, 6H), 2.26, 2.10 (two s of rotamers, 3H). ^{13}C NMR (101 MHz, $CDCl_3$): δ 155.39, 155.23, 155.18, 147.70, 147.63, 147.47, 147.29, 147.03, 141.61, 141.58, 141.52, 141.45, 140.55, 139.08, 137.94, 137.77, 135.86, 134.93, 134.77, 133.08, 130.33, 130.12, 129.97, 129.21, 129.14, 126.23, 125.87, 125.79, 125.48, 125.41, 125.15, 124.14, 123.96, 123.77, 122.74, 122.62, 122.55, 121.79, 119.43, 119.26, 114.65, 114.58, 114.21, 55.54, 55.40, 21.49, 20.28. Elemental analysis (calcd., found for $C_{61}H_{51}N_3O_4$): C (82.31, 82.19), H (5.78, 5.76), N (4.72, 4.87).

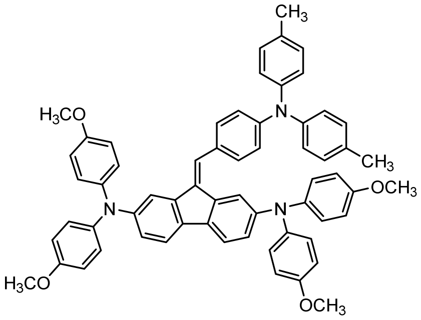

4-[{2,7-bis(4,4'-dimethoxydiphenylamino)-9*H*-fluoren-9-ylidene}methyl)-*N*,*N*-bis(4-methylphenyl)aniline (HTM4)

The mixture of **4** (1.21 g, 2 mmol), bis(4-methoxyphenyl)amine (1.38 g, 6 mmol), palladium acetate (0.009 g, 0.04 mmol), tri-tert-buthylphosphonium tetrafluoroborate (0.016 g, 0.054 mmol), sodium tert-butoxide (0.58 g, 6 mmol) in 13 ml of anhydrous toluene was refluxed for 52 hours under argon atmosphere. Afterwards, water was added and the extraction was done with ethyl acetate. The organic layer was dried over anhydrous Na_2SO_4, filtered, and the solvent was evaporated. The crude product was purified by column chromatography using 1:24 v/v tetrahydrofurane/*n*-hexane as an eluent to collect compound **HTM4** as a red solid. The product was precipitated from 20% solution of the solid residue in acetone which was poured while intensively stirring into a twentyfold excess of ethanol. Yield: 1.05 g (58%). ^1H NMR (400 MHz, $CDCl_3$): δ 7.59 (d, J = 2.1 Hz, 1H), 7.41-6.71 (m, 36H), 3.79 (s, 6H), 3.62 (s, 6H), 2.31 (s, 6H). ^{13}C NMR (101 MHz, $CDCl_3$): δ 155.33, 155.19, 147.85, 147.25, 146.97, 145.07, 141.63, 141.56, 141.09, 137.60, 134.95, 134.44, 132.82, 132.57, 130.28, 129.89, 129.32, 129.25, 127.41, 125.76, 125.40, 124.60, 122.67, 122.60, 122.42, 119.50, 119.37, 119.30, 116.88, 114.64, 114.54, 114.07, 55.53, 55.40, 20.89. Elemental analysis (calcd., found for $C_{62}H_{53}N_3O_4$): C (82.36, 82.21), H (5.91, 6.09), N (4.65, 4.46).

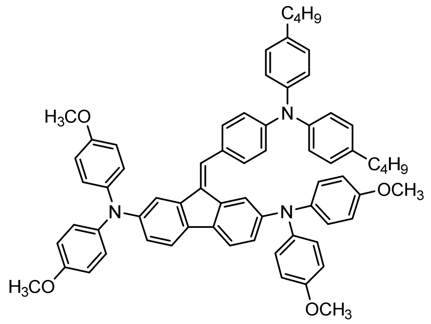

4-[{2,7-bis(4,4'-dimethoxydiphenylamino)-9*H*-fluoren-9-ylidene}methyl)-*N*,*N*-bis(4-butylphenyl)aniline (HTM5)

The mixture of **5** (1.38 g, 2 mmol), bis(4-methoxyphenyl)amine (1.38 g, 6 mmol), palladium acetate (0.009 g, 0.04 mmol), tri-tert-buthylphosphonium tetrafluoroborate (0.016 g, 0.054 mmol), sodium tert-butoxide (0.58 g, 6 mmol) in 14 ml of anhydrous toluene was refluxed for 52 hours under argon atmosphere. Afterwards, water was added and the extraction was done with ethyl acetate. The organic layer was dried over anhydrous Na_2SO_4, filtered, and the solvent was evaporated. The crude product was purified by column chromatography using 1:24 v/v tetrahydrofurane/*n*-hexane as an eluent to collect compound **HTM5** as a red solid. The product was precipitated from 20% solution of the solid residue in acetone which was poured while intensively stirring into a twentyfold excess of ethanol. Yield: 0.99 g (50%). ^{1}H NMR (400 MHz, $CDCl_3$): δ 7.56-6.71 (m, 35H), 3.79 (s, 6H), 3.62 (s, 6H), 2.56 (t, J = 7.3 Hz, 4H), 1.59 (quin, J = 7.3 Hz, 4H), 1.38 (sex, J = 7.3 Hz, 4H), 0.94 (t, J = 7.3 Hz, 6H). ^{13}C NMR (101 MHz, $CDCl_3$): δ 147.82, 145.20, 137.55, 130.19, 129.39, 129.10, 124.36, 122.77, 119.48, 114.53, 55.50, 55.37, 35.07, 33.71, 22.46, 14.00. Elemental analysis (calcd., found for $C_{68}H_{65}N_3O_4$): C (82.64, 82.46), H (6.63, 6.45), N (4.25, 4.07).

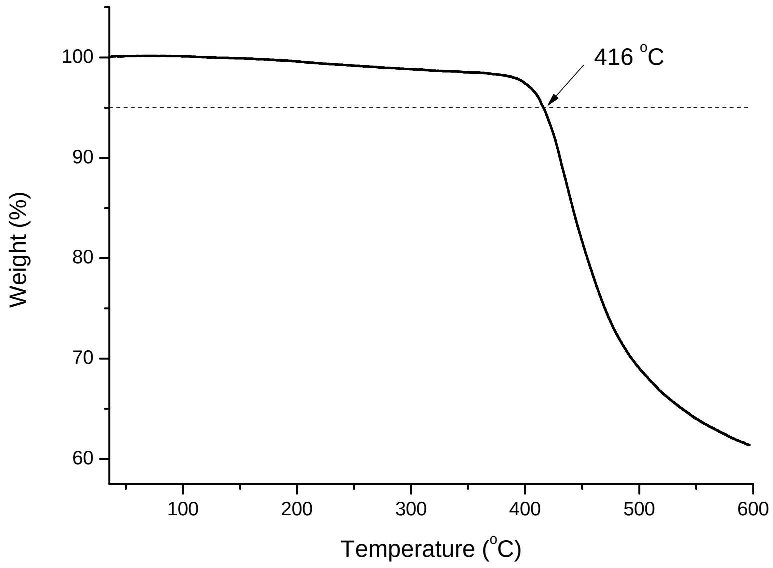

Figure S1. Thermogravimetric heating curve of **HTM1** (heating rate 10 °K min^{-1}).

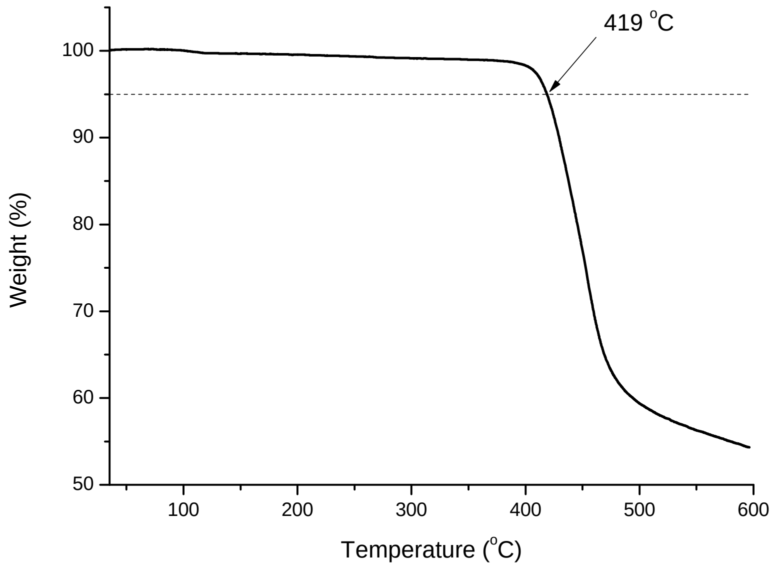

Figure S2. Thermogravimetric heating curve of **HTM2** (heating rate 10 °K min^{-1}).

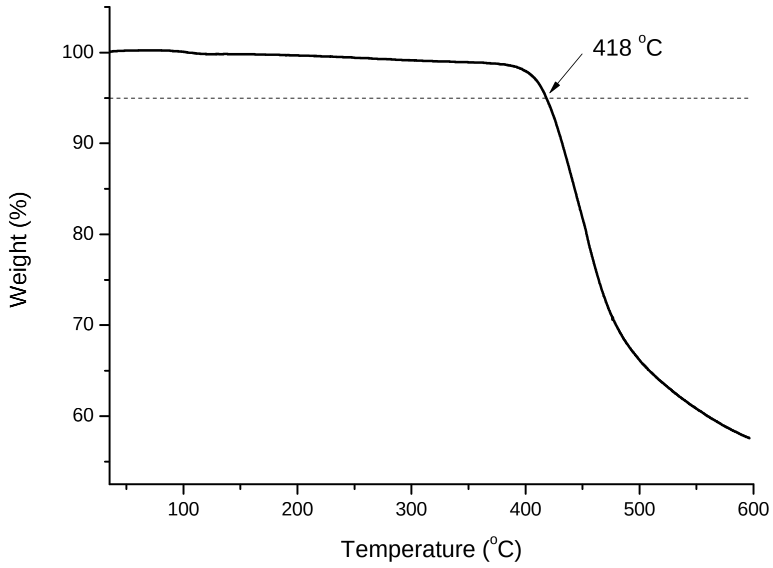

Figure S3. Thermogravimetric heating curve of **HTM3** (heating rate 10 °K min^{-1}).

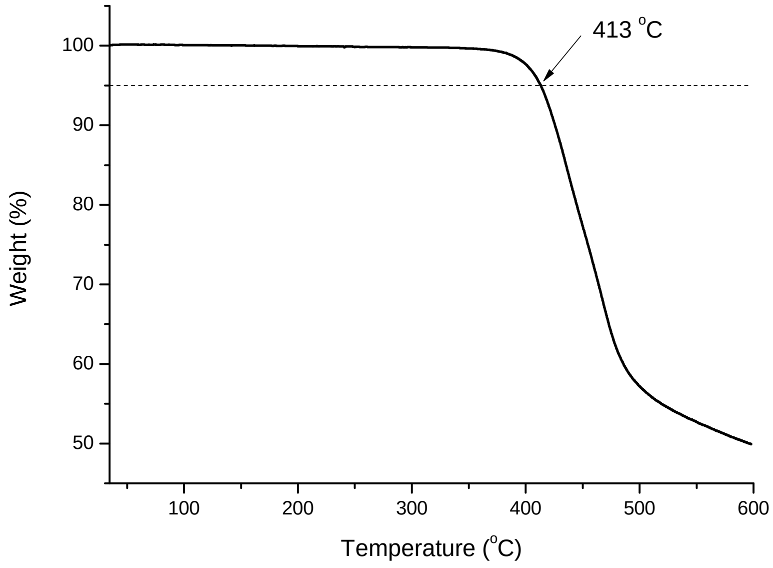

Figure S4. Thermogravimetric heating curve of **HTM5** (heating rate 10 °K min^{-1}).

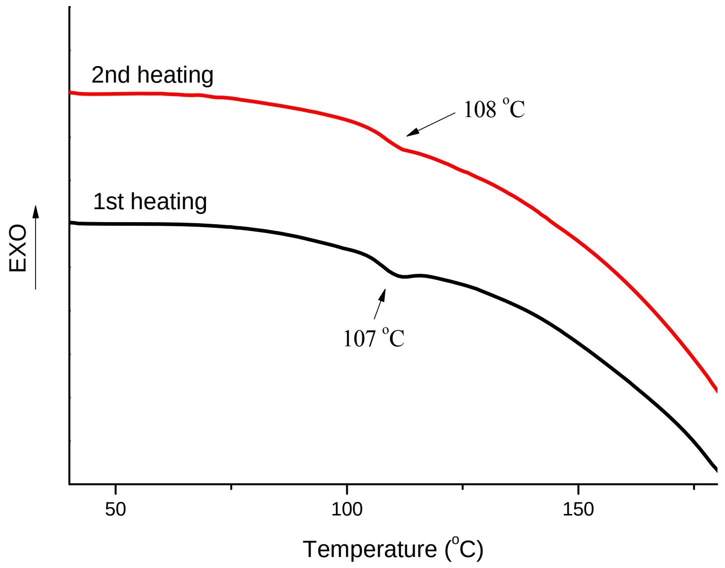

Figure S5. Differential scanning calorimetry first and second heating curves of **HTM1** (heating rate 10 °K min^{-1}).

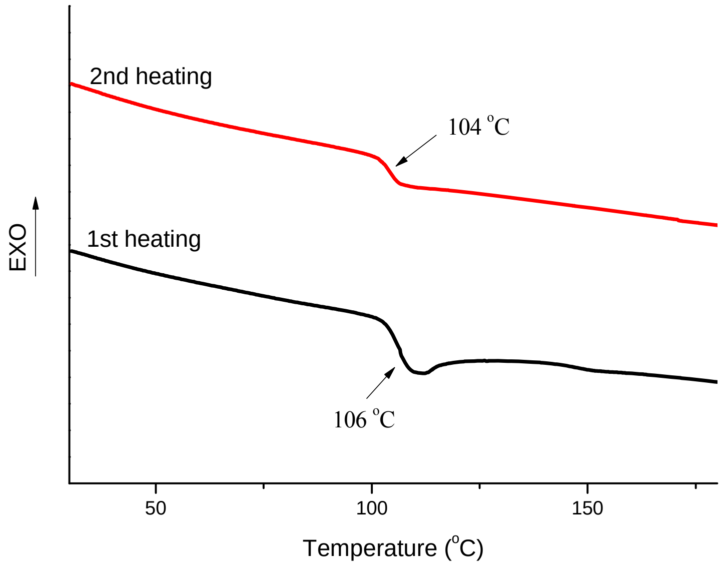

Figure S6. Differential scanning calorimetry first and second heating curves of **HTM2** (heating rate 10 °K min^{-1}).

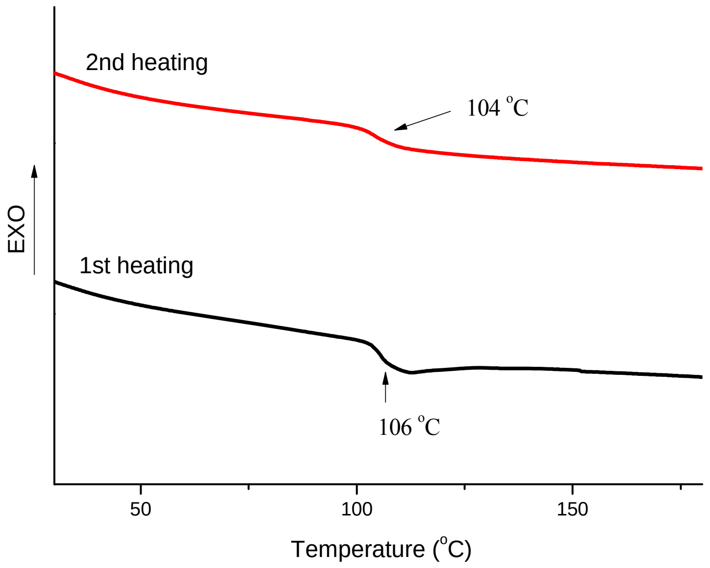

Figure S7. Differential scanning calorimetry first and second heating curves of **HTM3** (heating rate 10 °K min^{-1}).

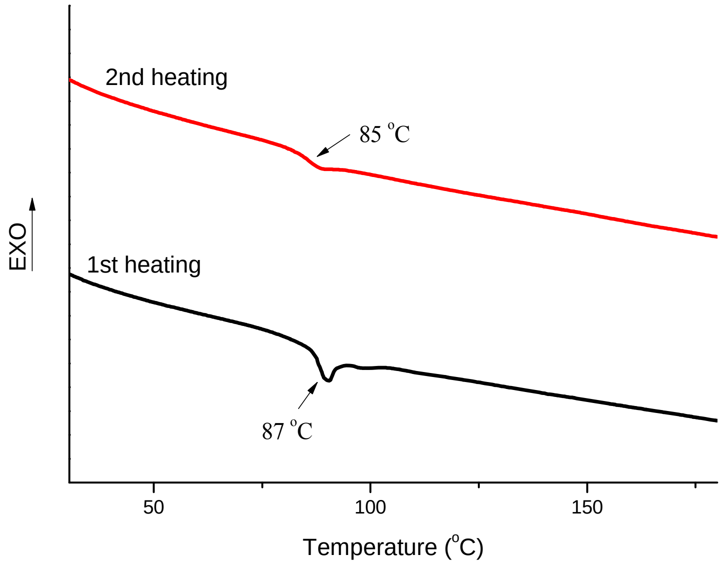

Figure S8. Differential scanning calorimetry first and second heating curves of **HTM5** (heating rate 10 °K min^{-1}).

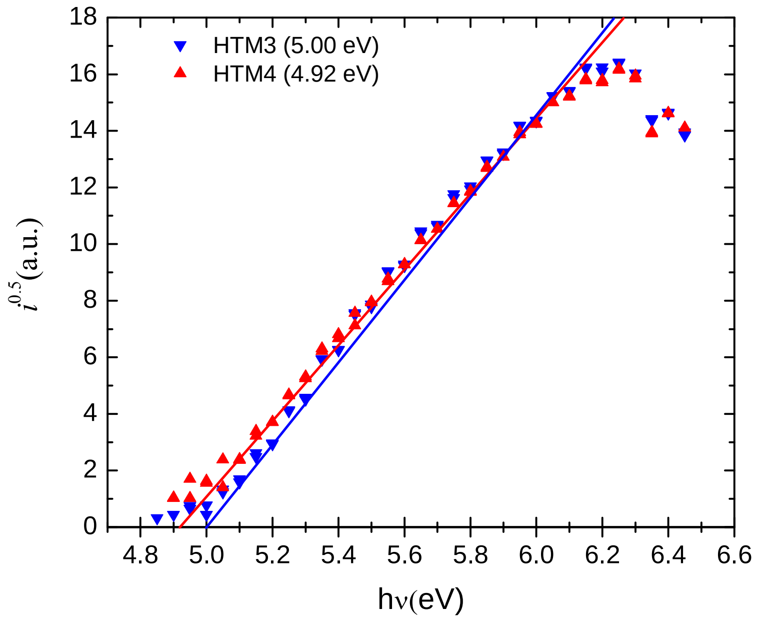

Figure S9. Photoemission in air spectra of **HTM3** and **HTM4**.

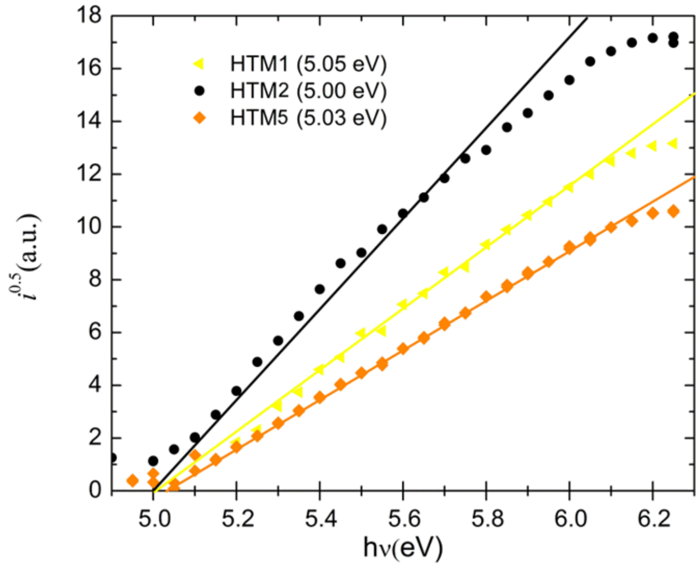

Figure S10. Photoemission in air spectra of **HTM1**, **HTM3** and **HTM6**.

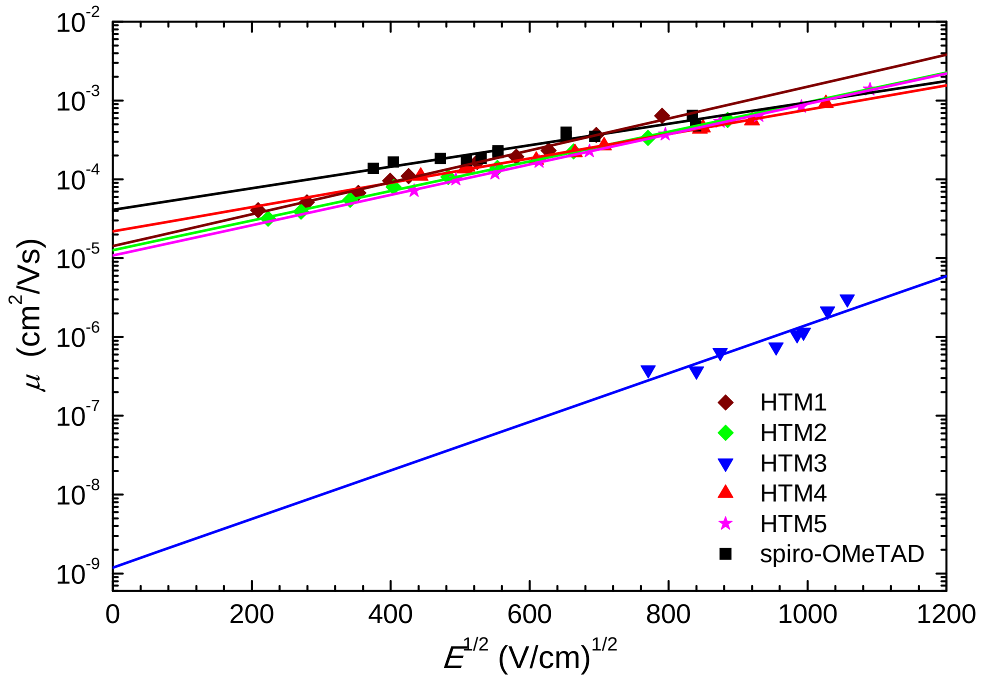

Figure S11. Hole drift mobility of **HTM1-5** and spiro-OMeTAD.

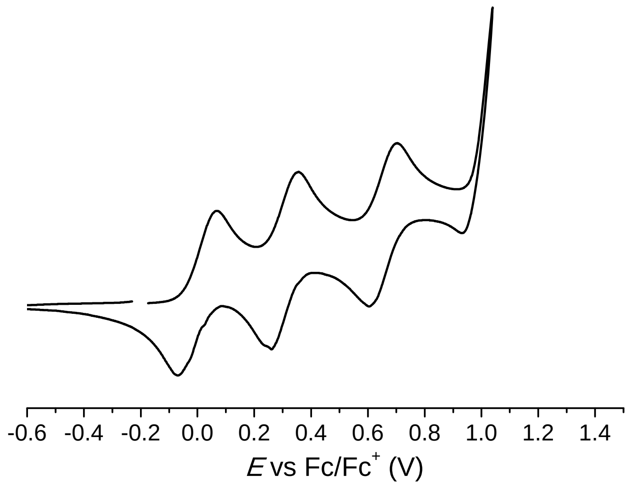

Figure S12. Oxidation waves of **HTM1** (scan rate = 50 mV·s^{-1}) in argon-purged dichloromethane solution.

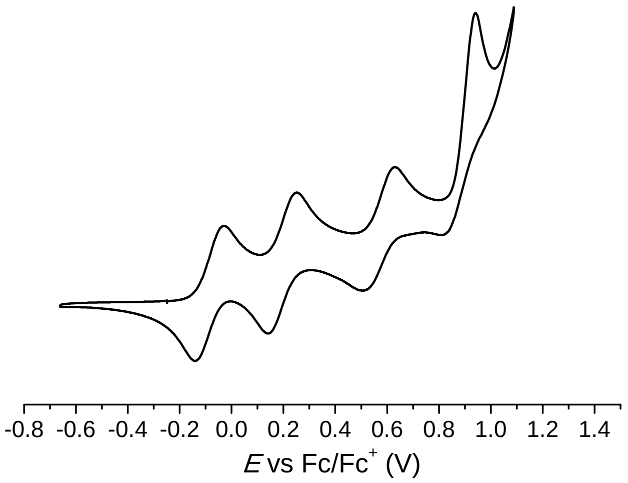

Figure S13. Oxidation waves of **HTM2** (scan rate = 50 mV·s^{-1}) in argon-purged dichloromethane solution.

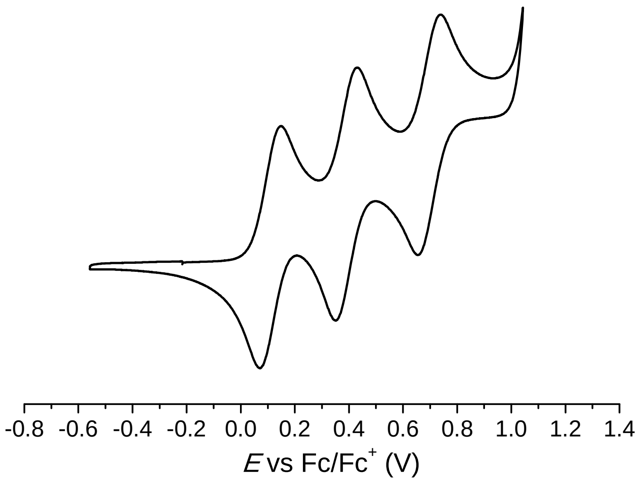

Figure S14. Oxidation waves of **HTM3** (scan rate = 50 mV·s^{-1}) in argon-purged dichloromethane solution.

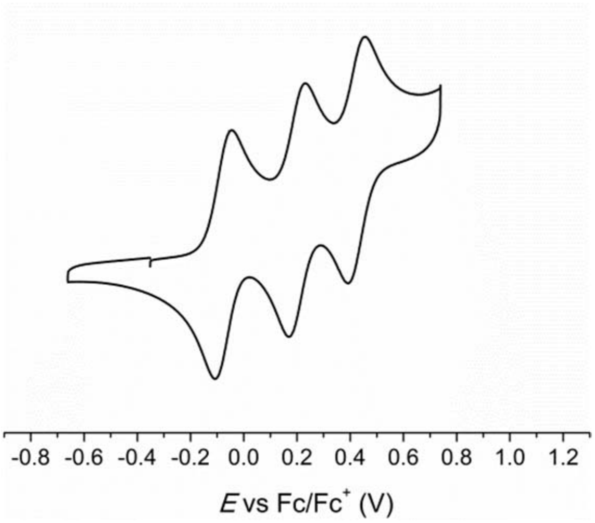

Figure S15. Oxidation waves of **HTM4** (scan rate = 50 mV·s^{-1}) in argon-purged dichloromethane solution

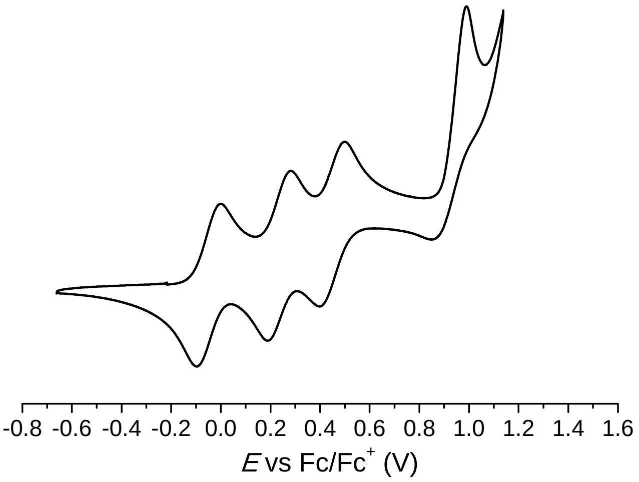

Figure S16. Oxidation waves of **HTM5** (scan rate = 50 mV·s^{-1}) in argon-purged dichloromethane solution.

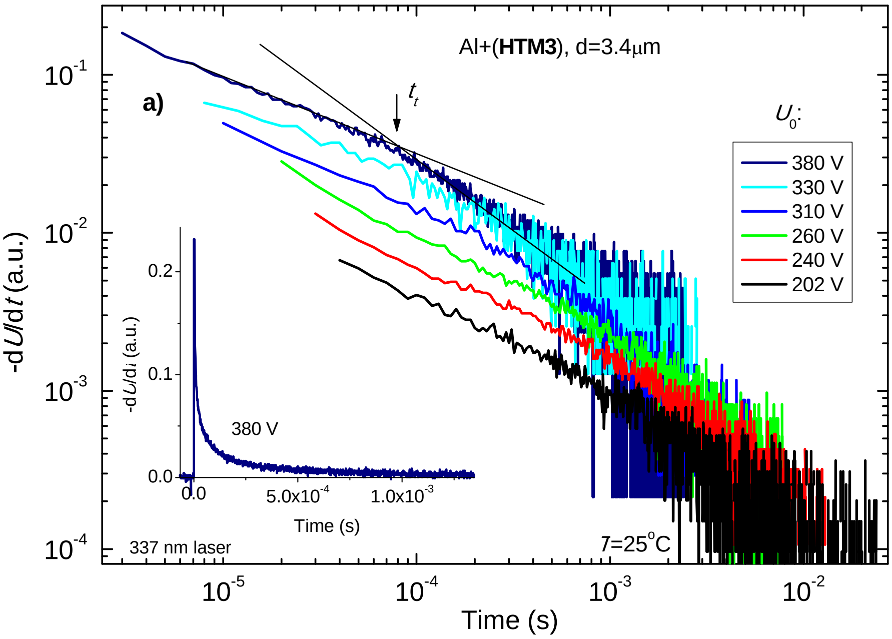

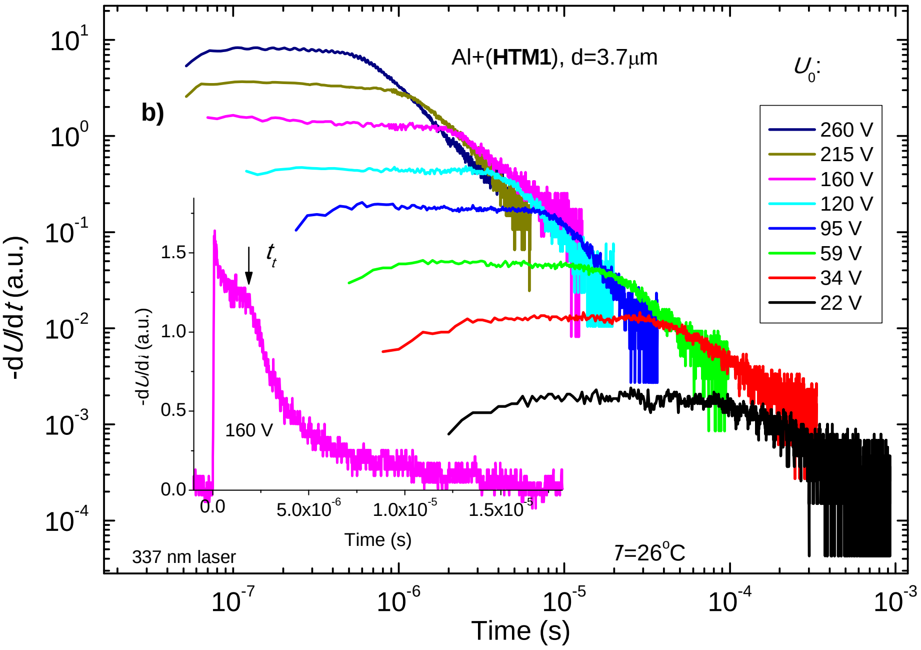

Figure S17. Transient photocurrents in the layers of **HTM3** (a) and **HTM1** (b) at different applied sample voltages, inserts show the one transient curve in linear plot.

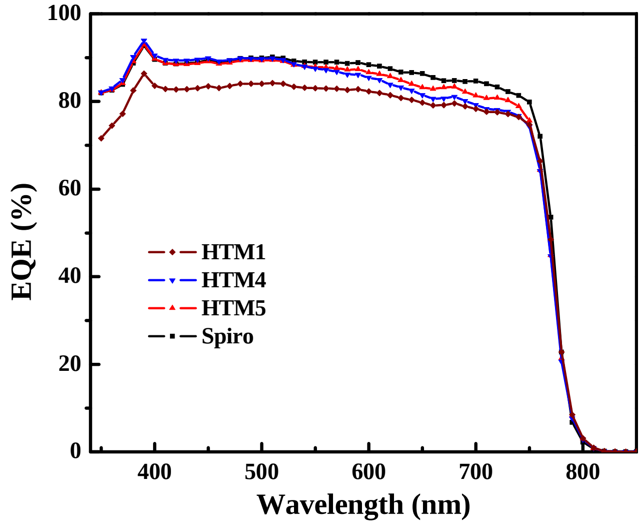

Figure S18. IPCE spectra of FTO/TiO_2/perovskite/**HTM**(**1, 4, 5**), spiro)/Au in perovskite solar cell.

Table S1. Short circuit current-density (J_{sc}), open-circuit voltage (V_{oc}), fill factor (FF) and power conversion efficiency (PCE) of the best performing spiro-OMeTAD, **HTM1–HTM5** devices on perovskite. Devices were masked with a metal aperture of 0.16 cm^2 to define the active area. No device preconditioning, such as light soaking or forward voltage bias applied for long time, was applied before starting the measurement.

Materials	J_{SC}, mA/cm^2	V_{OC}, mV	FF	PEC (%)
**HTM1**	19.075	1005	75.7	14.52
**HTM2**	17.526	1146	74.2	15.09
**HTM3**	13.371	915	70.7	9.15
**HTM4**	21.269	1052	75.0	16.79
**HTM5**	21.136	1029	75.7	16.45
**Spiro**	21.607	1092	75.3	17.88

References

[1] E. Miyamoto, Y. Yamaguchi, M. Yokoyama, *Electrophotography* **1989**, *28*, 364–370.

[2] M. Cordona, L. Ley, *Top. Appl. Phys.* **1978**, *26*, 1.

[3] S. M. Vaezi-Nejad, *Int. J. Electron.* **1987**, *62*, 361–384.

[4] Y. Archie, C. Chan, C. Juhasz, *Int. J. Electron.* **1987**, *62*, 625–632.
